# Supplementary material for: ESM-1 Overexpression is Involved in Increased Tumorigenesis of Radiotherapy-Resistant Breast Cancer Cells
Source: Cancers (Basel). 2020 May 26;12(6):1363. doi: 10.3390/cancers12061363 (PMC7352712; doi:10.3390/cancers12061363)
Supplement: Supplementary file 1 [file cancers-12-01363-s001.zip › cancers-787989 Supplementary Material.docx]

Supplementary Material: ESM-1 Overexpression is Involved in Increased Tumorigenesis of Radiotherapy-Resistant Breast Cancer Cells

Hana Jin, Trojan Rugira, Young Shin Ko, Sang Won Park, Seung Pil Yun and Hye Jung Kim

**Figure S1.** Whole blot image from Figure 1A.

**Figure S2.** Whole blot image from Figure 2A.

**Figure S3.** Whole blot image from Figure 3A.

**Figure S4.** Whole blot image from Figure 4A,C,E.

**Figure S5.** Whole blot image from Figure 5A,C,E.

**Figure S6.** Whole blot image from Figure 6A.

Table S1 (separate Excel file ): Intensity ratio of bands (*n* = 5).
